# Long-term treated HIV infection is associated with platelet mitochondrial dysfunction

**DOI:** 10.1038/s41598-021-85775-5

**Published:** 2021-03-18

**Authors:** Wouter A. van der Heijden, Lisa van de Wijer, Martin Jaeger, Karin Grintjes, Mihai G. Netea, Rolf T. Urbanus, Reinout van Crevel, Lambertus P. van den Heuvel, Maaike Brink, Richard J. Rodenburg, Philip G. de Groot, Andre J. van der Ven, Quirijn de Mast

**Affiliations:** 1 Department of Internal Medicine, Radboud Center for Infectious Diseases, Radboud University Medical Center, 6500 HB Nijmegen, The Netherlands; 2 Department for Genomics and Immunoregulation, Life and Medical Sciences Institute (LIMES), University of Bonn, Bonn, Germany; 3 Department of Clinical Chemistry and Hematology, University Medical Center Utrecht, Utrecht University, Utrecht, The Netherlands; 4 Department of Pediatrics, Radboud Center for Mitochondrial Medicine, Radboud University Medical Centre, Nijmegen, The Netherlands; 5 Translational Metabolic Laboratory, Department Laboratory Medicine, Radboud University Medical Centre, Nijmegen, The Netherlands; 6 Synapse Cardiovascular Research Institute Maastricht, Maastricht University Medical Center, Maastricht, The Netherlands

**Keywords:** Medical research, HIV infections, Cardiovascular diseases

## Abstract

HIV infection and antiretroviral therapy have been linked to mitochondrial dysfunction. The role of platelet mitochondrial dysfunction in thrombosis, immunoregulation and age-related diseases is increasingly appreciated. Here, we studied platelet mitochondrial DNA content (mtDNA_pl_) and mitochondrial function in people living with HIV (PLHIV) and related this to platelet function. In a cohort of 208 treated PLHIV and 56 uninfected controls, mtDNA_pl_ was quantified, as well as platelet activation, platelet agonist-induced reactivity and inflammation by circulating factors and flow cytometry. In a subgroup of participants, the metabolic activity of platelets was further studied by mitochondrial function tests and the Seahorse Flux Analyzer. PLHIV had significantly lower mtDNA_pl_ compared to controls (8.5 copies/platelet (IQR: 7.0–10.7) vs. 12.2 copies/platelet (IQR: 9.5–16.6); *p* < 0.001), also after correction for age, sex and BMI. Prior zidovudine-use (n = 46) was associated with a trend for lower mtDNA_pl_. PLHIV also had reduced ex vivo platelet reactivity and mean platelet volume compared to controls. MtDNA_pl_ correlated positively with both platelet parameters and correlated negatively with inflammatory marker sCD163. Mitochondrial function tests in a subgroup of participants confirmed the presence of platelet mitochondrial respiration defects. Platelet mitochondrial function is disturbed in PLHIV, which may contribute to platelet dysfunction and subsequent complications. Interventions targeting the preservation of normal platelet mitochondrial function may ultimately prove beneficial for PLHIV.

## Introduction

Mitochondrial dysfunction is a well-known phenomenon in people living with HIV (PLHIV), which has been linked with the use of nucleoside reverse transcriptase inhibitors (NRTIs)^1–5^. The main mechanism underlying NRTI toxicity is inhibition of mitochondrial DNA polymerase γ and increased oxidative stress, resulting in mitochondrial DNA (mtDNA) depletion^6–8^, and mitochondrial dysfunction at the tissue level^7,9^. These adverse effects were greatly reduced when the older NRTIs stavudine, zidovudine (AZT) and didanosine were replaced by the newer NRTIs tenofovir (TDF) and abacavir (ABC). However, these newer NRTIs may still impair mitochondrial function, albeit to a lesser degree^10–13^. More recently, mitochondrial dysfunction has also been reported in people living with HIV (PLHIV) naive for combined antiretroviral therapy (cART)^3,14–16^, including in elite-controllers ^17^. The factors responsible for cART-independent mtDNA depletion are less well defined and may involve persistent immune activation^18,19^. Mitochondrial dysfunction has been suggested to contribute to non-AIDS related co-morbidities such as cardiovascular diseases, diabetes, cancer and dementia in PLHIV^20,21^.

Platelets are the second most numerous blood cells that are, unlike red blood cells, equipped with mitochondria with mtDNA^22,23^. Human platelets lack a nucleus and mitochondria are essential in maintaining platelet health and lifespan, as recently reviewed^23^. MtDNA copy number is considered to reflect mitochondrial function^24^. Healthy platelets contain between 5 and 8 mitochondria, which serve important processes such as platelet activation, ATP production and platelet viability^25–29^. Platelets are traditionally known for their role in hemostasis, but an increasing body of evidence supports their role in key processes beyond hemostasis, including inflammation and immunoregulation^23^. With the importance of mitochondria in platelet metabolism, it is no surprise that the health consequences of abnormalities in platelet mitochondrial DNA and function has received increased attention, and platelet mtDNA has been proposed to serve as biomarker for different diseases^22,30–32^. Data on platelet function in PLHIV is contradictory, with some studies reporting increased ^33–37^, but other reduced agonist-induced platelet reactivity^28,38,39^.

We hypothesized that mitochondrial dysfunction in PLHIV is associated with reduced platelet mtDNA copies and platelet dysfunction. Here, we show that platelet mtDNA copies are lower in PLHIV on long-term cART and this was validated in a subgroup of PLHIV using mitochondrial functional assays.

## Results

### Cohort characteristics

Between December 2016 and February 2017, a total of 208 virally suppressed PLHIV on long-term cART and 56 healthy controls (sampled twice) were concurrently enrolled. Baseline characteristics are shown in Table 1. PLHIV were older compared to controls (52 years (IQR: 45.8–59.0) vs 30 years (IQR: 25.8–53), respectively, *p* < 0.001). Median duration of cART use was 6.6 years (IQR: 4.2–11.9).

**Table 1:** Baseline characteristics.

	PLHIV (n = 208)	Healthy controls (n = 56)
Sex (% Female)	17 (8.2)	22 (39.2)*
Age (years, median [IQR])	52.0 [45.8, 59.0]	30.0 [25.8–53.0]*
BMI (median [IQR])	24.1 [22.0, 26.0]	23.8 [21.5–25.6]
HIV infection duration (years, median [IQR])	8.5 [5.0, 14.2]	
**Way of transmission (%)**		
Heterosexual	8 (3.8)	
IDU	3 (1.4)	
MSM	158 (76.0)	
Other/unknown	39 (18.8)	
CD4 nadir (median [IQR])	250.0 [135.0, 362.5]	
CD4 count (median [IQR])	660.0 [480.0, 812.5]	
Undetectable HIV load, n (%)	208 (100)	
CD4/CD8 ratio (median [IQR])	0.8 [0.6, 1.1]	
cART duration (years; median [IQR])	6.6 [4.1, 11.8]	
**cART regimen**		
NRTI-use (%)	200 (96.2)	
NtRTI-use (%)	97 (46.6)	
NNRTI-use (%)	61 (29.3)	
PI-use (%)	32 (15.4)	
Maraviroc-use (%)	3 (1.4)	
INSTI-use (%)	140 (67.3)	
ABC (%)	93 (44.7)	
DTG (%)	86 (41.3)	
EVG (%)	15 (7.2)	
RAL (%)	38 (18.3)	
Smoking (%)	59 (28.4)	
Pack years (median [IQR])	13.8 [0.0, 28.0]	
Hypercholesterolemia (%)	56 (26.9)	
Hypertension (%)	40 (19.2)	
Diabetes Mellitus (%)	9 (4.3)	
No cardiovascular risk factors (%)	50 (24.0)	
Statins (%)	56 (26.9)	
Aspirin (%)	18 (8.7)	
Metformin (%)	9 (4.3)	

*BMI* body mass index, *cART* combination antiretroviral therapy, *NRTI* nucleoside reverse transcriptase inhibitor, *NtRTI* nucleotide reverse transcriptase inhibitor, *NNRTI* non-nucleoside reverse transcriptase inhibitor, *PI* protease inhibitor, *INSTI* integrase inhibitor, *ABC* abacavir, *DTG* dolutegravir, *EVG* elvitegravir, *RAL* raltegravir.

*Significantly different between cohorts.

### Platelet mtDNA copies are reduced in PLHIV

**Figure 1:**
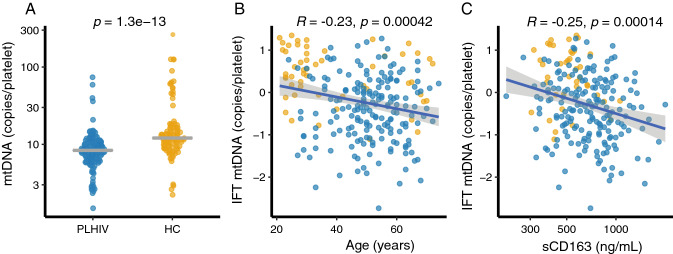
Platelet mtDNA content. Mitochondrial DNA content in platelets (mtDNA_pl_) was determined using qPCR after normalization for platelet count in isolated platelets. (**A**) Dotplot showing mtDNA copies per platelet in people living with HIV (PLHIV) and controls (HC); the line indicates median and error bars the interquartile range (IQR). Student’s t-test was used after inverse rank-based normalization. (**B**) Normalized (inverse rank based) mtDNA_pl_ versus age. (**C**) Normalized mtDNA_pl_ versus soluble CD163 (a macrophage scavenger receptor). Correlations were analyzed using Pearson’s correlation coefficients.

Platelet mtDNA copies (mtDNA_pl_) were significantly lower in PLHIV compared to healthy controls (Fig. 1A; median 8.5 copies/platelet (plt) (IQR 7.0–10.7) vs 12.2 copies/plt (IQR: 9.5–16.6), *p* < 0.001). mtDNA_pl_ copies correlated inversely with age (Fig. 1B; Pearson’s R = − 0.24, p < 0.001). MtDNA_pl_ remained significantly associated with HIV status after correction for age, sex and body-mass index (BMI) in a linear regression model (B = 0.77 SE: 0.14, *P* < 0.0001). When analysis was restricted to individuals of 40 years and above (Supplemental Table S1; PLHIV: n = 173 vs HC: n = 22), mtDNA_pl_ remained significantly lower in PLHIV compared to controls (Supplemental Fig. S1; PLHIV: 8.4 copies/plt (IQR: 6.6–10.2) vs HC: 10.5 copies/plt (IQR: 8.9–14.7), *p* = 0.001). Similarly, the differences in mtDNA_pl_ between PLHIV and controls remained when analysis was restricted to males only (Supplemental Table S2; PLHIV: 8.5 copies/plt (IQR: 6.6–10.7) vs HC: 11.6 copies/plt (IQR: 9.3–14.7), *p* < 0.001). MtDNA_pl_ levels were neither associated with duration of cART nor with current CD4 count, CD4 nadir or CD4/CD8 ratio in univariate analysis (Supplemental Table S3). Furthermore, no differences in mtDNA_pl_ were found between PI, NNRTI and INSTI-based regimens (*all p* > 0.1; Supplemental Fig. S2).

Although abacavir (ABC)-use has been shown to affect platelet function^40^, current ABC use (*p* = 0.89) or cumulative ABC exposure (in days) were not associated with mtDNA_pl_ copies (Supplemental Fig. S2). Conversely, PLHIV with prior zidovudine-use (prior AZT-use, n = 46/184; 25%) showed a trend towards lower copies of mtDNA_pl_ (AZT-use: 7.8 copies/plt [IQR: 6.0, 9.4] vs never-AZT: 8.6 copies/plt [IQR: 6.9, 10.8], *p* = 0.055). After correcting for age in a linear regression model, a non-significant negative correlation remained (B: − 0.07 (− 0.15 to 0.01, p = 0.069). Neither cumulative days on AZT (median 2069 days; IQR 792–3180 days; R = − 0.2, *p* = 0.12), nor total NRTI exposure (median 4520 days; IQR: 2734–7955; R = 0.05) correlated with mtDNA-use. Use of metformin (p = 0.079) or antihypertensive drugs (p = 0.061) showed a tendency for a lower mtDNA_pl_ copies, whereas use of statins (*p* = 0.74) or acetylsalicylic acid (*p* = 0.21) did not (Supplemental Fig. S3).

Mitochondrial dysfunction is associated with inflammatory diseases such as atherosclerosis and sepsis in HIV-negative individuals^22^. Hence, we explored the association of known markers of persisting immune activation, a known driver of non-AIDS related comorbidities^20,21^, with mtDNA_pl_ copy number. Overall, we observed higher plasma concentrations of immune activation markers soluble CD14 (sCD14), soluble CD163 (sCD163) and high-sensitive CRP (hsCRP) in PLHIV than in controls (Table 2). Among participants older than 40 years, only sCD163 remained significantly different between groups (Supplemental Table S1; HIV 741 ng/mL (IQR: 547–906) vs controls 517 ng/mL (IQR: 443–684), *p* = 0.01). sCD163 correlated negatively with mtDNA_pl_ copies (R = − 0.23, *p* < 0.001, Fig. 1C), whereas sCD14 (R = − 0.073, *p* = 0.27) and hsCRP (R: − 0.087, *p* = 0.2) did not (Supplemental Fig. S4).

**Table 2:** MPV: mean platelet volume.

Platelet indices	PLHIV (n = 208)	Healthy controls (n = 56)	p-value
Platelet count (10^9^/L)	260 [200, 310]	270 [210, 320]	0.126
MPV (fL)	10.1 [9.7, 10.7]	10.8 [10.3, 11.3]	< 0.001
IPF (%)	3.3 [2.5, 4.6]	3.7 [2.8, 5.9]	0.018
Unstimulated fibrinogen binding (MFI)	2.0 [1.6, 2.2]	2.1 [1.6, 2.4]	0.072
Unstimulated P-selectin expression (MFI)	2.7 [2.1, 3.2]	2.6 [2.2, 3.0]	0.158
Plasma CCL5 (ng/ml)	2.66 [1.64, 4.29]	2.61 [1.69, 4.18]	0.841
Plasma CXCL4 (ng/mL)	570.0 [290.0, 723.3]	492.9 [306.1, 839.5]	0.201
Plasma CXCL7 (ng/mL)	289.4 [180.3, 465.1]	306.6 [194.2, 609.8]	0.192
sCD14 (ng/mL)	2139.6 [1778.2, 2661.5]	1789.0 [1502.7, 2071.6]	< 0.001
sCD163 (ng/mL)	716.5 [528.7, 899.4]	517.3 [410.7, 578.1]	< 0.01
hsCRP (ng/mL)	1423.3 [608.8, 2726.3]	651.2 [205.9, 1179.2]	< 0.001

Unstimulated platelet aggregation measured as Fibrinogen binding by flowcytometry in median fluorescence intensity (MFI). Unstimulated platelet degranulation measured as P-selectin expression by flow cytometry by MFI.

Data were analyzed using Mann–Whitney *U* test.

*IPF* immature platelet fraction as a percentage of platelet count (Sysmex, Kobe, Japan); *sCD14* serum levels of CD14, a marker of monocyte activation; *sCD163* serum levels of CD163, a monocyte- and macrophage-specific scavenger receptor; *hsCRP* high sensitive C-reactive protein.

### Platelet mitochondrial dysfunction in PLHIV

**Figure 2:**
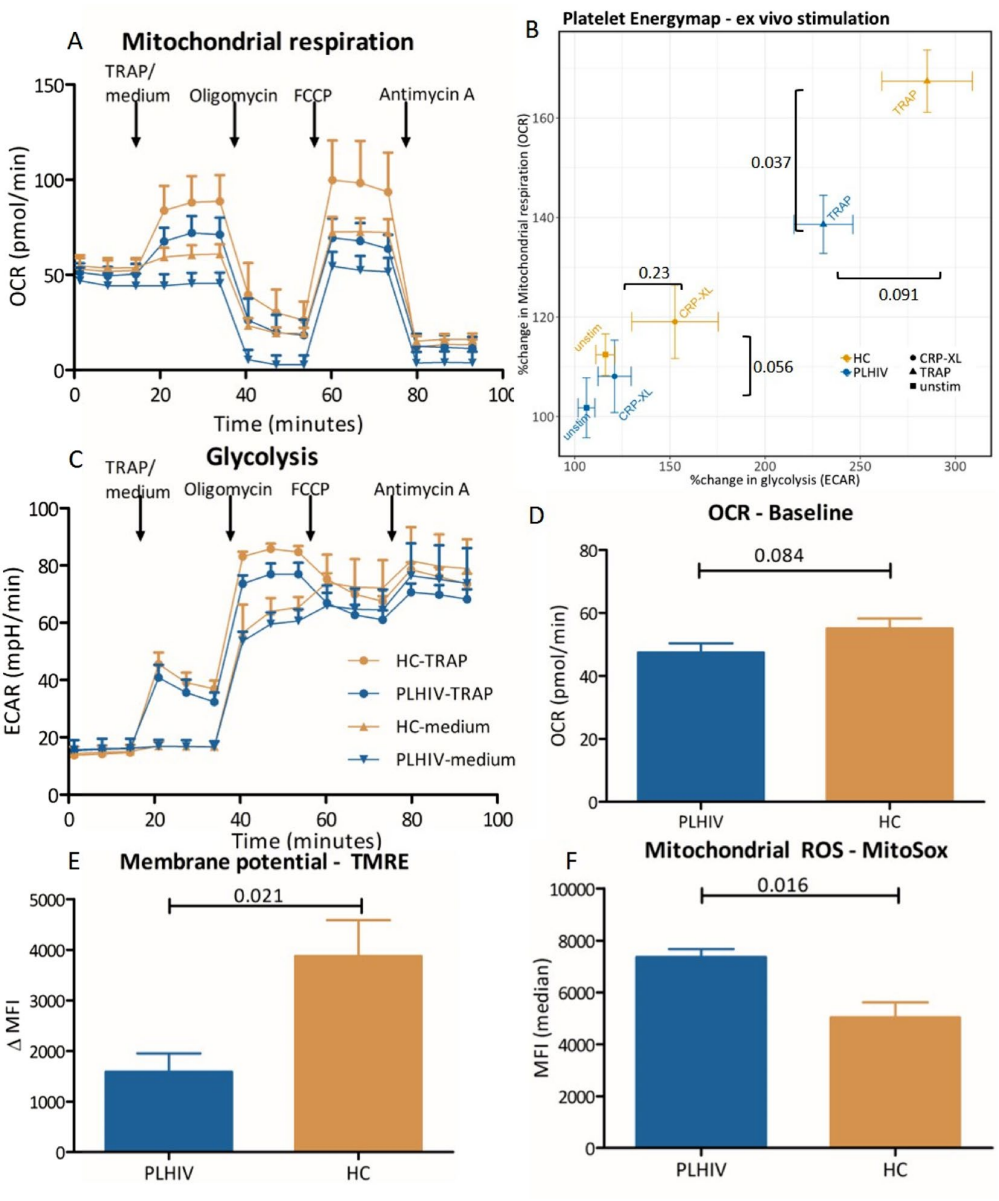
Platelet energy phenotype determined by the Seahorse extracellular flux analyzer and mitochondrial membrane potential and ROS production. Samples were measured with five replicates, 1 × 10^7^ washed platelets per well. Five age-and sex-matched donors per group. (**A**) Real-time mitochondrial respiration depicted as oxygen consumption rate (OCR) at 18 timepoints. Four agonists or inhibitors were added in the following order: (1) Thrombin Receptor Activator Peptide-6 (TRAP-6; 50 µM) or medium causing platelet activation; (2) Oligomycin which inhibits cellular ATP production; (3) Carbonyl cyanide-4-(trifluoromethoxy) phenylhydrazone (FCCP), an uncoupling agent causing maximum oxygen consumption through complex IV, and (4) Antimycin A which inhibits all mitochondrial respiration (CRP-XL can be found in Supplemental Fig. 9). (**B**) Energymap showing the change in mitochondrial respiration (as change in OCR) and glycolysis (extracellular acidification rate (ECAR) from basal conditions after ex vivo stimulation with platelet agonists. ((**A**)) Depicts the real-time ECAR after (1) medium or TRAP-6 (2) oligomycine, (3) FCCP and (4) antimycin A. Samples were measured with five replicates, 1 × 10^7^ washed platelets per well. Five age-and sex-matched donors per group. (**D**) Basal OCR as mean of first three measurements. (**E**) Membrane potential was determined using Tetramethylrhodamine ethyl ester (TMRE) staining with the uncoupler FCCP disrupting mitochondrial membrane potential as a negative control for every sample. Data depicted as delta geometric mean fluorescence intensity (MFI). (**F**) Mitochondrial ROS production with MitoSox staining in MFI. Data are depicted as mean ± standard error of the mean (SEM) and analyzed using the unpaired student’s T-test.

Energy demand for platelet ATP production and other metabolic processes that are essential for platelet activation is met by the combined actions of glycolysis and mitochondrial OXPHOS^41^. To validate our findings that the lower platelet mtDNA content is associated with platelet mitochondrial dysfunction, and to investigate platelet glycolysis activity, we assessed the metabolic activity of washed platelets of five PLHIV and five age-sex matched controls to assess membrane potential (Δψ_m_), mitochondrial superoxide production (ROS_m_) and real-time glycolysis and mitochondrial respiration using the Seahorse Extracellular Flux Analyzer^41^. Platelet Δψ_m_, as assessed by Tetramethylrhodamine ethyl ester (TMRE) fluorescence, was lower in PLHIV compared to controls (1669 ΔMFI (IQR: 911–2233) vs. 4545 ΔMFI (IQR: 2249–5168) respectively, *p* = 0.02; Fig. 2E, t-test). In line with reduced Δψ_m_, there was a small increase in ROS_m_ in PLHIV at basal conditions (Fig. 2F; 7654 MFI (IQR: 6777–7798) vs 4956 MFI (IQR: 3840–6284), *p* = 0.007, t-test). Next, using the Seahorse Extracellular Flux Analyzer, mitochondrial respiration and glycolytic capacity were investigated by assessing the oxygen consumption rate (OCR; measure for mitochondrial respiration/OXPHOS) and the acidification rate (ECAR; measure for glycolysis). There was a trend towards lower baseline platelet mitochondrial respiration (OCR in pmol/min) in PLHIV compared to controls (Fig. 2D; 47.4 pmol/min (SD: 11.3) vs 55.1 pmol/min (SD: 12.2), p = 0.084). Additionally, PLHIV had a smaller increase in OCR after ex vivo platelet stimulation with both CRP-XL (108% (SD: 16) vs 119% (SD: 16), *p* = 0.056, Fig. 2B and Supplemental Fig. S5) and Thrombin receptor activating peptide (TRAP; 139% (SD: 14) vs 167% (SD: 13), *p* = 0.037; Fig. 2A–C) compared to matched controls.

**Figure 3:**
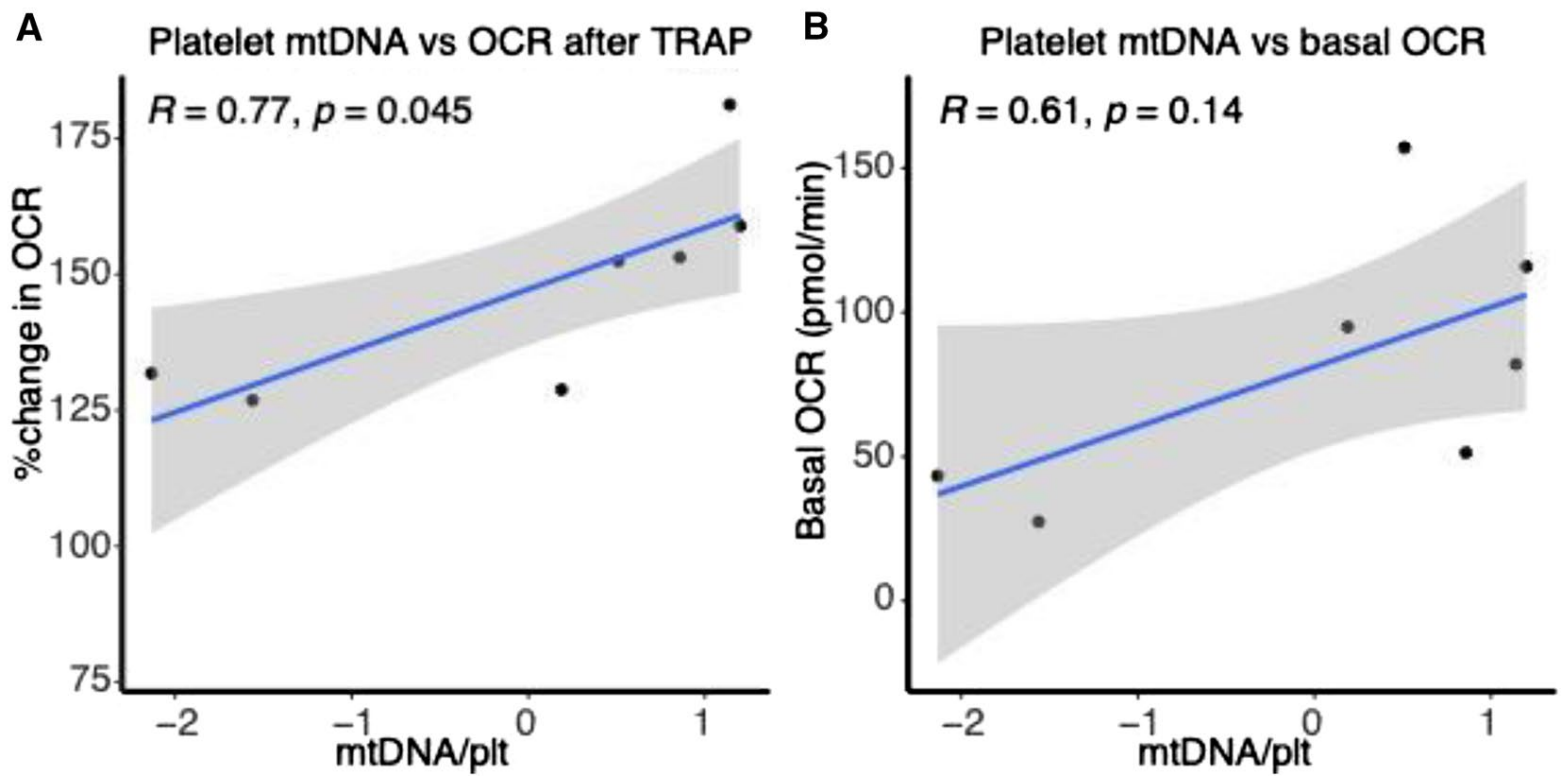
Platelet mitochondrial DNA vs mitochondrial respiration measured by Oxygen consumption rate (OCR in pmol/min). (**A**) Correlation plot showing platelet mitochondrial DNA (mtDNA) with change in oxygen consumption rate (mitochondrial respiration) measured by Seahorse extracellular flux analyzer after ex vivo stimulation with thrombin (TRAP 50 μM). mtDNA was normalized using inverse rank-based transformation and Pearson’s correlation coefficient is shown. (**B**) mtDNA in platelets vs basal OCR in pmol/min (an average of first three measurements). OCR was measured in triplo and for 5 PLHIV and 2 controls both mtDNA in platelets and real-time measurements of mitochondrial respiration were available and included in the analysis.

The fraction of the maximal mitochondrial capacity (maximal OCR after stimulation with the uncoupling agent FCCP) used after ex vivo platelet stimulation with TRAP was high in both groups (Supplemental Fig. S6d; p > 0.1). This suggests that maximal mitochondrial capacity is a limiting factor in platelet activation. Conversely, there was no difference between groups in ECAR (glycolysis) at baseline (Fig. 2B; HIV: 16.0 mpH/min (SD: 3.4) vs HC: 14.7 mpH/min (SD: 2.4), *p* > 0.1) or after ex vivo stimulation. The increase in mitochondrial respiration (OCR) after ex vivo stimulation significantly correlated with mtDNA_pl_ (R = 0.77, p = 0.045, n = 7, Fig. 3A) and a similar trend was found for basal mitochondrial respiration of platelets (R = 0.61, p = 0.14, n = 7, Fig. 3B). Taken together, our data suggest that the lower platelet mtDNA in PLHIV in associated with a concurrent reduction in mitochondrial respiration capacity (OXPHOS) without a compensatory increase in glycolysis.

### HIV infection is associated with platelet dysfunction

Next, we explored the possible consequences of lower mtDNA_pl_ and mitochondrial dysfunction for platelet parameters and function. Platelet counts were similar between PLHIV and healthy controls (Table 2), but PLHIV had smaller platelets (mean platelet volume: PLHIV:10.1 fL (IQR: 9.7:10.7) vs 10.8 fL (IQR: 10.3–11.3), *p* < 0.0001), as well as a lower immature platelet fraction (IPF; a marker for freshly released platelets from the bone marrow) compared to controls (Table 2; 3.3% (IQR: 2.5–4.6) vs 3.7% (IQR: 2.8–5.9) respectively, *p* = 0.018). Platelet size (mean platelet volume) correlated positively with mtDNA_pl_ (Fig. 5C).

**Figure 4:**
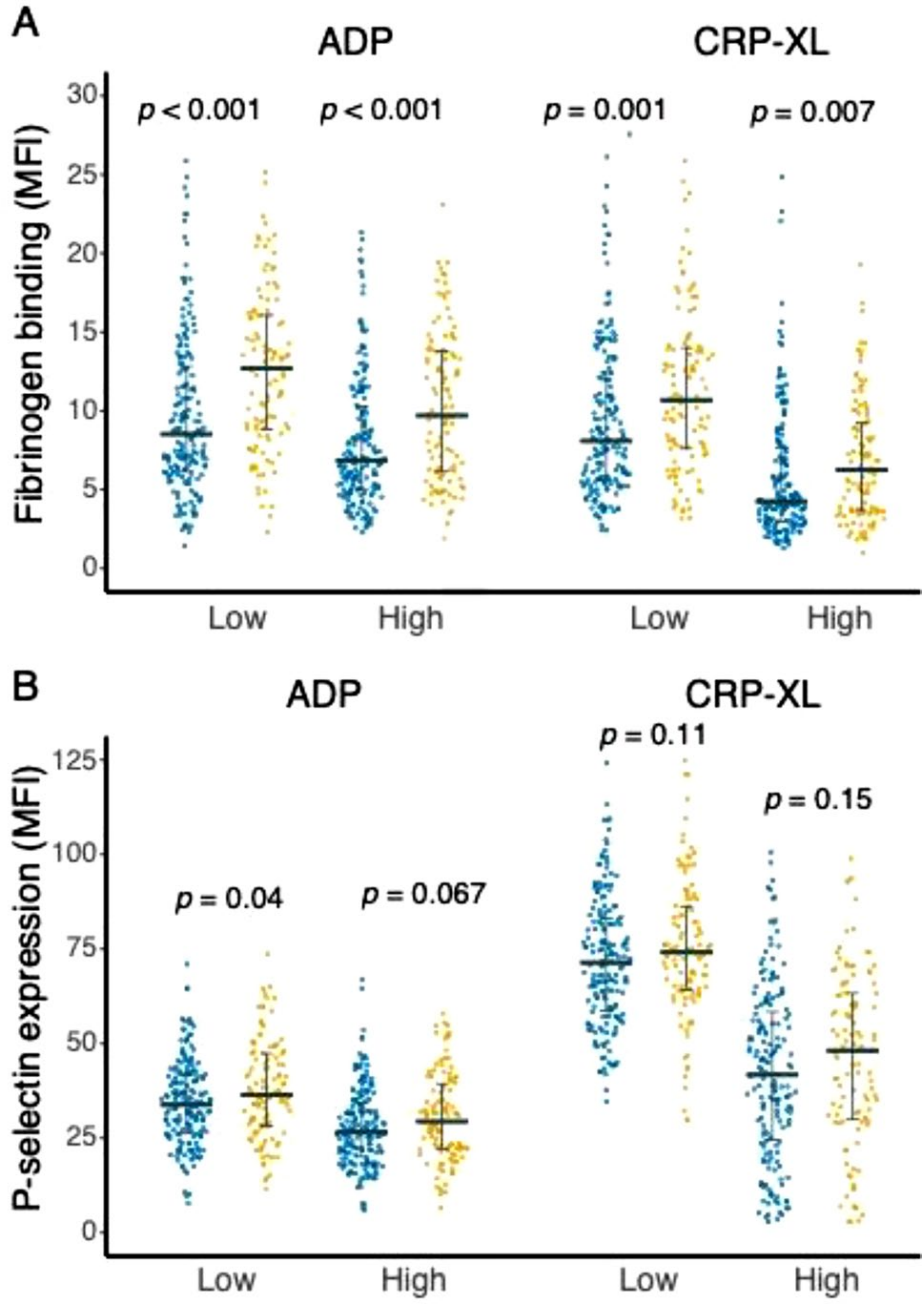
Platelet reactivity after collagen (CRP-XL) and ADP stimulation. (**A**) Platelet fibrinogen binding is depicted as mean fluorescence intensity (MFI) after ex vivo stimulation with platelet agonist collagen-related peptide (CRP-XL; 33 ng/mL; 625 ng/mL) and adenosine diphosphate (ADP; 1.2 μM, 125 μM). (**B**) Platelet degranulation measured by P-selectin expression is depicted as mean fluorescence intensity (MFI) after ex vivo stimulation with CRP-XL (33 ng/mL; 625 ng/mL) and ADP (1.2 μM, 125 μM). Data are shown as dotplot with error bars median and interquartile range (IQR). Data were analyzed using unpaired Student’s T-test. People living with HIV (PLHIV); healthy controls (HC).

Next, platelet activation and function and function were determined using multiple methods. First, plasma markers of in vivo platelet activation (chemokines released from alpha-granules; CCL5, CXCL4, CXCL7) were comparable between PLHIV and controls (Table 2). Second, using flow cytometry, the activation status of circulating platelets, as well as their reactivity to ex vivo stimulation by platelet agonists was assessed. In unstimulated platelets, the expression of the alpha-granule marker P-selectin (measure of platelet degranulation) and the binding of fibrinogen to the activated integrin αIIbβ3 (measure of aggregation; Table 2) were also similar across groups. When analysis was restricted to individuals above 40 years of age or male only, unstimulated platelet activation was lower in PLHIV compared to controls (Supplemental Tables S1, S2). In line with this observation, fibrinogen binding to αIIbβ3 in response to stimulation by adenosine diphosphate (ADP)- and collagen related peptide (CRP-XL) was reduced in PLHIV-induced) (Fig. 4A). Differences in P-selectin reactivity across the groups were smaller with only a significant difference with a high dose (125 µM) of ADP stimulation showed a significant difference between PLHIV and controls (Fig. 4B). We observed no correlations with cART regimens containing either NNRTI, PI or INSTI-use as well as ABC and platelet reactivity indices (all *P* > 0.15). In addition, persistent immune activation did not correlate with platelet indices in this cohort (Supplemental Table S4). In summary, these data show that platelet reactivity, and especially αIIbβ3 activation is reduced in PLHIV.

### Association of mtDNA_pl_ with platelet function

**Figure 5:**
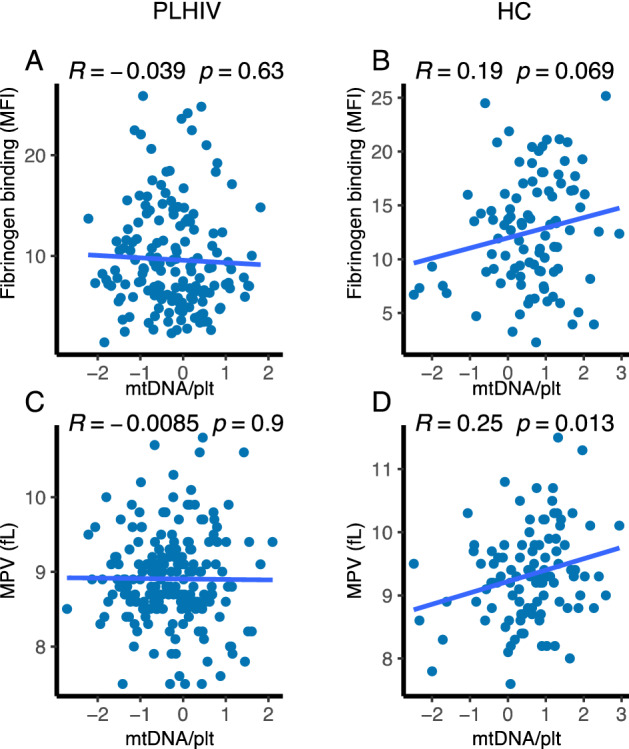
Association of platelet mitochondrial DNA with platelet reactivity and mean platelet volume (MPV). (**A**, **B**) correlation of normalized mitochondrial DNA content in platelets (mtDNA_pl_) and the binding of fibrinogen to the activated integrin αIIbβ3 after ex vivo platelet stimulation with ADP (125uM) in PLHIV (**A**) and healthy controls (**B**). (**C**, **D**) correlation of normalized mitochondrial DNA content in platelets (mtDNA_pl_) and MPV in PLHIV (**C**) and healthy controls (**D**). All correlations were analyzed using Pearson’s correlation coefficients.

Next, given the role of platelet mitochondria in platelet function, we assessed associations of mtDNA_pl_ with platelet reactivity in PLHIV and controls. MtDNA_pl_ copies did neither correlate with ADP-induced P-selectin expression, nor with binding of fibrinogen to platelets or mean platelet volume (MPV) in PLHIV (Fig. 5A,B). In controls, MtDNA_pl_ copies were significantly correlated with MPV (Fig. 5C) and a positive trend was observed with fibrinogen binding (Fig. 5D).

To further explore the link between platelet reactivity and platelet activation, we performed a principal component analysis (PCA) to summarize both platelet activation (plasma markers of platelet activation and unstimulated P-selectin expression and fibrinogen binding) and platelet reactivity (P-selectin expression and fibrinogen binding after ex vivo ADP and CRP-XL stimulation). This PCA showed that Principal component (PC) 1 mainly represented platelet reactivity (P-selectin expression and fibrinogen binding after stimulation) whereas PC2 mainly represented in vivo platelet activation (plasma markers and unstimulated P-selectin expression and fibrinogen binding; Supplemental Fig. S7a,b). We used these derivatives to correlate mtDNA_pl_ with platelet reactivity (coordinates on PC1) and platelet activation (coordinates on PC2). mtDNA_pl_ correlated with platelet reactivity (Supplemental Fig. S7c; PC1 of platelet parameters vs mtDNA_pl_, R = 0.14, p = P = 0.024), but not with in vivo platelet activation (Supplemental Fig. S7d; PC2 of platelet parameters vs mtDNA_pl_, R = 0.05, P = 0.41). These data suggest that mtDNA depletion is associated with platelet dysfunction with a reduced platelet reactivity capacity, but not with increased platelet activation status.

Whereas mitochondrial dysfunction can result in platelet dysfunction, platelet activation itself may also contribute to loss of mitochondria from platelets through the formation of platelet microparticles (PMP) formation^42^. We therefore studied PMP in a subgroup of 20 PLHIV and age-sex matched controls. No differences in total PMP number (Supplemental Fig. S8a), nor PMPs containing mitochondria (Supplemental Fig. S8b) were observed across both groups.

## Discussion

The present data show that PLHIV on long-term cART have reduced platelet mitochondrial content (mtDNA_pl_) which was associated with platelet mitochondrial dysfunction and reduced energy supply. Platelet mitochondria play a key role in platelet metabolism, ATP production and platelet activation and lifespan, and we propose that the observed abnormalities in mtDNA_pl_ and platelet mitochondrial function contribute to platelet dysfunction in PLHIV.

The literature on platelet function in cART treated individuals is contradictory, with some studies reporting increased platelet reactivity^33–37^, while others reporting reduced reactivity^28,38,39^. This heterogeneity in study results may be partly explained by differences in the characteristics of study participants, including the enrolment of PLHIV with detectable plasma HIV-RNA, the degree of persistent immune activation, cART regimens and timing of treatment initiation, factors that all have changed considerably over the years. In accordance with our present findings, Mesquita et al.^28^ recently reported decreased platelet reactivity and platelet mitochondrial dysfunction in 36 PLHIV on stable cART. In contrast to our findings, platelets in the PLHIV in their cohort exhibited increased P-selectin expression. In the present study, not only platelet P-selectin expression, but also soluble markers of platelet activation were similar in PLHIV and controls. In addition, PLHIV exhibited a lower immature platelet fraction and a similar number of mitochondria-containing platelet microparticles. Together, these findings argue against excessive platelet activation being primarily responsible for reduced platelet reactivity in PLHIV. Whether mitochondrial depletion contributes to the observed platelet dysfunction in PLHIV remains uncertain. A recent study reported that chemotherapy-associated platelet hyporeactivity was caused by mitochondrial dysfunction and subsequent reduction in mitochondrial respiration^27^. Consistent with these observations, the lower platelet mtDNA content in PLHIV was associated with a reduction in mitochondrial respiration capacity, which may negatively impact platelet reactivity. In our study, however, mtDNA_pl_ levels in PLHIV did not correlate with ex vivo platelet reactivity, whereas a positive trend was observed in HIV uninfected controls. The fact that all PLHIV exhibited reduced mtDNA_pl_ with little variation in the absolute values may explain the absence of a correlation with platelet reactivity measures. Future studies focusing on platelet dysfunction should incorporate mitochondrial dysfunction to corroborate our findings.

Age, history of zidovudine (AZT)-use and innate immune activation were all associated with decreased mtDNA_pl_. NRTI-use is a well-known cause of mitochondrial dysfunction and mtDNA depletion^6–8^. We found that prior AZT-use was a possible risk factor for reduced mtDNA_pl_, a trend that remained after correcting for age and CD4 nadir, whereas total duration of cART-use or duration of HIV infection were not. As platelets are short-lived, it is conceivable that mitochondrial mass is reduced during thrombopoiesis and that the known bone marrow toxicity of AZT ^1^ is still present even after switching to newer NRTIs such as TDF or ABC. Even though these NRTIs are known to have lower mitochondrial toxicity^1^, it is unclear whether long-term treatment does not exert any cumulative reduction in mtDNA_pl_ too. While ABC-use has been linked to platelet perturbations in multiple studies^43–45^, others could not confirm ABC associated platelet dysfunction^35,46^. In our study, neither mtDNA_pl_ content nor platelet function were associated with current or prior ABC-use. Unfortunately, we could not dissect the link between the mtDNA_pl_, overall NRTI exposure and duration of HIV infection itself, as exposure to NRTIs was high in the total study group. Still, as mtDNA_pl_ content was lower in PLHIV than in controls, it is plausible that both NRTIs and HIV itself exert long-term changes in mitochondrial function^2^. This possible long-term NRTI effect on mitochondrial function in platelets supports recent efforts to implement NRTI sparing regimens as viable treatment options for long-term HIV treatment^47^. It would be interesting to assess mtDNA_pl_ content, as well as platelet function, in PLHIV who are switched to a NRTI sparing regimen.

Mitochondrial dysfunction and depletion have been associated with many diseases such as dementia, neuropsychiatric diseases, and cardiovascular diseases^22,23,30,48^. In PLHIV, these (non-AIDS related) co-morbidities have also been linked to persistent inflammation^20,21^. In our cohort, we indeed found increased levels of hsCRP, sCD14 and sCD163, but only the latter parameter was associated with mtDNA levels in platelets. Importantly, sCD163 was shown to be independently correlated with overall mortality in PLHIV and the incidence of non-AIDS related comorbidities^49,50^.

Targeting mitochondrial dysfunction and inflammation may help reduce excess mortality and morbidity that is associated with HIV infection, even when treated successfully with cART^22,27^. Reducing inflammation, besides reducing NRTI exposure, could indirectly reduce oxidative stress and mitochondrial dysfunction while treatment with ROS scavengers may also have beneficial effects in reducing non-AIDS related co-morbidities.

Multiple methods of mtDNA quantification have been used in whole blood or peripheral blood mononuclear cell (PBMC) fractions in HIV and other diseases^14,32^. However, different methods of quantification, and heterogeneity of the cell composition of whole blood or PBMCs may hamper its interpretation^32,51^. It is conceivable that platelet mtDNA content could mirror mitochondrial toxicity in other cell types as well. As a single cell-type source of mtDNA, platelet mtDNA may indeed serve as a possible biomarker for mitochondrial toxicity and non-AIDS related comorbidities associated with mitochondrial dysfunction, such as neurocognitive impairment and cardiovascular disease^22^.

Our study has limitations associated with its cross-sectional design. Even though sample size was large enough to explore the link between inflammation, cART-use, mtDNA_pl_ and platelet function, it lacked power to confirm a possible link between mtDNA_pl_ levels and clinical outcomes such as non-AIDS co-morbidities and NRTI-related adverse events. Although we observed reduced oxygen consumption in individuals with reduced mtDNA_pl_, the overall reduction of mtDNA_pl_ in PLHIV prevented to investigate functional consequences of mtDNA_pl_ depletion in PLHIV. Furthermore, we did not perform immunofluorescence confocal or transmission electron microscopy experiments in the current study. In addition, age and sex differed substantially between cohorts, with an effect of age on mtDNA content in the uninfected cohort. We explored multiple methods to account for these differences using adjusted models and subgroup analyses. The subgroup of above > 40 years revealed a significant difference between PLHIV and controls supporting the independent correlation of HIV infection in the age-sex adjusted model. Finally, our study included mostly Caucasian men limiting generalization of the findings to women and non-Caucasians.

In conclusion, PLHIV under long-term cART have reduced platelet mtDNA content and abnormalities in platelet mitochondrial respiration, which may possibly contribute to platelet dysfunction. Given the key role of platelets and mitochondria in the pathophysiology of long-term complications of HIV, interventions targeting platelet mitochondria, such as introducing NRTI sparing regimens, should be considered.

## Methods

### Patient selection

This cross-sectional, single center, prospective study was performed at the Radboud university medical center, a tertiary teaching hospital in The Netherlands. This study is part of the Human Functional Genomics Project (HFGP; www.humanfunctionalgenomicsproject.org) and was conducted in accordance with the Declaration of Helsinki after approval of the ethics committee (CMO Arnhem-Nijmegen, The Netherlands; NL42561.091.12, 2012/550). No animal experiments were performed in the current study. Adult HIV-1-infected individuals receiving cART for at least six months were included after providing written informed consent. Other inclusion criteria were a suppressed viral load (< 200 copies/mL). Exclusion criteria included, use of P_2_Y_12_ receptor antagonists (platelet inhibitor), an active hepatitis B or C infection and/or signs of other active intercurrent infection other than HIV-1 (e.g. fever in last week or antibiotic-use in last 4 weeks). Healthy controls were concurrently included throughout the duration of inclusion of PLHIV. Exclusion criteria were use of medication (excluding oral contraceptives or paracetamol) and/or signs of an active infection in the last month. Clinical data was collected by extracting data from electronic medical record (Epic Systems, Verona, WI, USA). History of cART use was extracted from the Dutch HIV registry (Stichting HIV-monitoring). In a separate validation experiment for mitochondrial function, five virally suppressed male PLHIV (45–60 years) who were not using statins or acetylsalicylic acid (ASA), were enrolled together with five age and sex matched controls.

### Platelet count and function

Platelet count and parameters were determined using an automated hematology analyzer (Sysmex, Kobe, Japan). Platelet reactivity was determined in citrated whole blood (3.2% sodium citrate, Becton Dickinson, Franklin Lakes, NJ, USA) using a flow cytometry based assay as previously described between 1 and 3 h after blood collection^52^. Platelets were ex vivo stimulated with ADP (1.2 and 125 μM; Sigma-Aldrich, Zwijndrecht, The Netherlands) and CRP-XL (a kind gift from Prof. Farndale, Cambridge, UK) for 20 min at room temperature. Platelets were stained using anti-CD61 (Beckman Coulter, Brea. CA, USA), anti-P-selectin (Biolegend, San Diego, CA, USA) and anti-fibrinogen (DAKO, Santa Clara, CA) antibodies and fixated in 0.2% paraformaldehyde. Platelets were identified based on Size (FSC), granularity (SSC) and their expression of CD61. Degranulation was determined as the membrane expression of α-granule protein P-selectin and platelet aggregation was quantified as the amount of fibrinogen binding to the activated integrin αIIbβ3. Platelet reactivity was measured on a FC500 flow cytometer (Beckman Coulter, Brea, USA). Data were extracted using Kaluza 2.1 (Beckman Coulter), normalized against quality controls to ensure measurement stability and are expressed as median fluorescence intensity (MFI). A gating strategy is provided in Supplemental Fig. S1.

### Platelet isolation

Platelet rich plasma (PRP) was obtained from citrated plasma (Vacutainer, Beckton-Dickinson) after centrifugation 156*g* for 15 min without brake at room temperature (RT). Samples were processed within 2 h of blood collection. Platelet count in PRP was measured using an automated hematology analyser (Sysmex, Kobe, Japan). Washed platelets were obtained as previously described^53^. In short, PRP was supplemented with acid citrate dextrose (10%) and prostaglandin I_2_ and washed twice with Hepes tyrode’s buffer using centrifugation (330*g*, 20 min).

### Platelet microparticles

Peripheral blood was centrifuged at 1000*g* for 5 min. Plasma was then centrifuged at 1500*g* for 20 min to obtain platelet poor plasma (PPP). 1 mL of PPP was centrifuged for 30 min at 20,000*g* to pellet microparticles (MPs). The MP pellet washed once in 500 µL calcium-free HBS complemented with 0.2% bovine serum albumin (pH 7.3) by 45 min centrifugation at 20,000*g*. MPs were labeled with MitoTracker Deep Red (200 nM; Invitrogen, Breda, The Netherlands) in HBS at room temperature containing calcium and subsequently stained with anti-CD61, anti-CD62p, Annexin-V (all Biolegend), anti-CD45 and anti-CD41 (both Beckman Coulter). MPs were analyzed using a Cytoflex flow cytometer (Beckman Coulter) including the sensitive violet Side Scatter (405 nM; VSSC) and FSC for detection of ultrasmall particles (1 µm)^54^. Platelet MPs (PMPs) were selected based on aforementioned markers. Counting beads (Sphero Nano fluorescent, Spherotech, Fulda, Germany) of different sizes were included for reference to correct for concentration and size. All solutions for PMP isolation and staining were centrifuged at 20,000*g* for 20 min to remove (fluorescent) aggregates. A gating strategy is provided in Supplemental Fig. S2.

### mtDNA quantification and mitochondrial function

After platelet count measurement of every PRP sample, 500 µL PRP was pelleted by centrifugation (2000*g*, 10 min at RT without brake) and lysed using Triton-X100 0.1% for mtDNA quantification. MtDNA_pl_ was measured by real time Quantitative PCR using Mitotox Quickscan (Primagen, Amsterdam, Netherlands), Advanced SYBR green Super mix (Bio-rad, Hercules, CA, USA) and CFX96 Real time Detection System (Bio-Rad) according to manufacturer’s instructions. The kit includes primers and calibrators for quantification. A calibration curve with known mtDNA concentration was concurrently measured on every plate to ensure stability and quantification. A dilution curve with PRP showed a high correlation coefficient for mtDNA and platelet concentration. mtDNA copies per platelet (mtDNA_pl_) are calculated by dividing mtDNA copy number per well by platelet count in 500 µL of PRP. Contamination of leukocyte and erythrocyte was low < 1:10,000. Samples with low platelet counts in PRP (below 50 × 10^12^/mL) and erythrocyte/leukocyte contamination were excluded (n = 2). Mitochondrial respiration was quantified using the 96-well format Seahorse extracellular flux analyzer (Agilent, MA, USA). Washed platelets were plated to 60,000 platelets/μL and seeded onto Cell-Tak coated XF96 microplates (Corning, Corning, NY, USA). A mitochondrial stress test was performed as described eslewhere^41^ using oligomycin (1 μM, ATP synthase inhibitor (complex V), reducing mitochondrial respiration), Carbonyl cyanide-4-(trifluoromethoxy) phenylhydrazone (FCCP; 1 μM, uncoupling agent causing maximum mitochondrial respiration), antimycin A (2.5 μM, Complex III inhibitor inhibiting mitochondrial respiration) and 2-deoxy-d-glucose (2DG; 40 mM, inhibits glycolysis) after ex vivo stimulation (all Sigma). Oxygen consumption rate (OCR) and extracellular acidification rate (ECAR) were determined before and after injection of platelet agonists. Samples were measured in five replicates with three measurements after every injection. For mitochondrial respiration measurements using Seahorse extracellular flux analyzer, significant outliers were excluded from analysis if measurement was 2× SD below the mean value in every timepoint with a maximum of one of the five replicates.

Mitochondrial membrane potential was determined using Tetramethylrhodamine ethyl ester (TMRE, Sigma; 100 nM at 37 °C for 20 min). A negative control was generated for every sample using FCCP (1 μM at 37 °C for 10 min). Mitochondrial reactive oxygen species (ROS_m_) were detected using the cationic probe MitoSOX Red (Invitrogen, Carlsbad, CA, USA (2.5 μM at 37 °C for 30 min). Both probes were quantified in CD61+ cells using a Cytoflex flow cytometer (Beckman Coulter).

### Plasma markers

Concentrations of three plasma markers of platelet activation: chemokine (C–X–C motif) ligand 4 (CXCL4 also known as platelet factor 4), CXCL7 (also known as beta-thromboglobulin) and chemokine (C–C motif) ligand (CCL5; also known as RANTES) were determined by ELISA (R and D systems, Minneapolis, USA) according to the manufacturer’s instructions in citrated PPP^52^. In addition, markers of persistent inflammation, high-sensitive C-reactive protein (hsCRP), sCD163 and sCD14, were measured using ELISA (Quantikine, R and D systems) according to the manufacturer’s instructions in EDTA plasma.

### Statistical analyses

Data were analyzed by independent T-test or Mann–Whitney U test. Pearson’s correlations coefficient was used for univariate correlation analyses, unless otherwise stated. An inverse rank-based normalization was performed for non-normal data (e.g. mtDNA in platelets). A multivariate linear regression model was used to correct for age, body mass index (BMI) and sex. Several sensitivity analyses using subgroups (males only and above 40 years) were performed to explore for possible confounding. Principal component analysis (PCA) was performed using singular value decomposition to summarize platelet function and correlate with mtDNA_pl_. cART-use was calculated as days on a certain drug, cumulative use of multiple drugs of the same class were combined. R studio (CRAN project) and Graphpad Prism version 5.03 were used for analyses.

## Supplementary Information

Supplementary Information.
