# Supplementary material for: Long-term treated HIV infection is associated with platelet mitochondrial dysfunction
Source: Sci Rep. 2021 Mar 18;11:6246. doi: 10.1038/s41598-021-85775-5 (PMC7973809; doi:10.1038/s41598-021-85775-5)
Supplement: Supplementary file 1 — Supplementary Information. [file 41598_2021_85775_MOESM1_ESM.docx]

**Supplemental material**

**Longterm treated HIV infection is associated with platelet mitochondrial dysfunction**

| Platelet indices | 200HIV | 50FG (n=22) | p -value |
| --- | --- | --- | --- |
| Platelet count (10^9/L) | 2.6 [2.0, 3.1] | 2.8 [2.4, 3.0] | 0.483 |
| MPV (fL) | 10.1 [9.7, 10.5] | 10.9 [10.3, 11.4] | **0.007** |
| IPF (%) | 3.2 [2.4, 4.5] | 5.9 [3.4, 9.4] | **0.008** |
| Baseline fibrinogen binding (MFI) | 2.0 [1.6, 2.2] | 2.2 [2.1, 2.3] | **0.002** |
| Baseline P-selectin expression (MFI) | 2.6 [2.0, 3.2] | 2.9 [2.8, 3.1] | **0.028** |
| CCL5 (ng/ml) | 2.66 [1.63, 4.23] | 2.07 [1.60, 3.19] | 0.245 |
| Platelet factor 4 (ng/ml) | 452.5 [294.5, 715.2] | 324.0 [258.3, 462.4] | 0.164 |
| CXCL7 (ng/ml) | 288.3 [180.9, 455.1] | 213.9 [189.1, 260.3] | 0.173 |
| sCD14 (ng/ml) | 2207.4 [1787.8, 2677.2] | 1952.2 [1741.9, 2134.7] | 0.062 |
| sCD163 (ng/ml) | 741.6 [547.3, 905.6] | 516.8 [442.7, 684.2] | **0.01** |
| hsCRP (ng/ml) | 1493.4 [608.4, 3051.2] | 812.6 [382.2, 1671.0] | 0.073 |

*Wouter A. van der Heijden^1^, Lisa Van de Wijer^1^, Martin Jaeger^1^, Karin Grintjes^1^, Mihai G. Netea^1 2^, Rolf T. Urbanus^3^, Reinout van Crevel^1^, Lambertus P. van den Heuvel^4^, Maaike Brink^5^, Richard J Rodenburg^4^, Philip G. de Groot^1 5^, Andre J. van der Ven^1^ , Quirijn de Mast^1^*

**Supplemental table 1 Platelet hematology indices, platelet activation parameters and markers of inflammation in individuals age >40 years.** MPV: mean platelet volume. IPF: immature platelet fraction as a percentage of platelet count. (Sysmex, Kobe, Japan). Baseline platelet aggregation measured as Fibrinogen binding by flowcytometry in median fluorescence intensity (MFI). Baseline platelet degranulation measured as P-selectin expression by flow cytometry by MFI. sCD14: Serum levels of CD14, a marker of monocyte activation. sCD163: Serum levels of CD163, a monocyte- and macrophage-specific scavenger receptor, hsCRP: high sensitive C-reactive protein. Data depicted as median [interquartile range] and analyzed using Mann–Whitney

| Platelet indices | 200HIV (n=191) | 50FG (n=34) | p -value |
| --- | --- | --- | --- |
| Platelet count (10^9/L) | 260 [200, 310] | 260 [220, 310] | 0.483 |
| MPV (fL) | 10.1 [9.7, 10.5] | 10.9 [10.3, 11.4] | **0.007** |
| IPF (%) | 3.3 [2.6, 4.7] | 5.0 [3.2, 7.0] | **0.007** |
| Baseline fibrinogen binding (MFI) | 2.0 [1.6, 2.2] | 2.2 [2.0, 2.4] | **<0.001** |
| Baseline P-selectin expression (MFI) | 2.7 [2.1, 3.2] | 3.0 [2.8, 3.2] | **0.008** |
| CCL5 (ng/ml) | 2.66 [1.63, 4.23] | 2.07 [1.60, 3.19] | 0.503 |
| Platelet factor 4 (ng/ml) | 458.4 [295.3, 726.1] | 494.0 [333.1, 858.2] | 0.39 |
| CXCL7 (ng/ml) | 290.4 [187.0, 452.8] | 317.1 [214.4, 542.5] | 0.466 |
| sCD14 (ng/ml) | 2155.2 [1780.7, 2672.7] | 1851.0 [1614.1, 2172.7] | **0.006** |
| sCD163 (ng/ml) | 700.2 [523.9, 900.0] | 532.1 [418.3, 620.5] | **0.002** |
| hsCRP (ng/ml) | 1451.7 [608.4, 2702.7] | 675.9 [166.5, 1309.3] | **0.003** |

**Supplemental table 2 Platelet hematology indices, platelet activation parameters and markers of inflammation in males only.** MPV: mean platelet volume. IPF: immature platelet fraction as a percentage of platelet count. (Sysmex, Kobe, Japan). Baseline platelet aggregation measured as Fibrinogen binding by flowcytometry in median fluorescence intensity (MFI). Baseline platelet degranulation measured as P-selectin expression by flow cytometry by MFI. sCD14: Serum levels of CD14, a marker of monocyte activation. sCD163: Serum levels of CD163, a monocyte- and macrophage-specific scavenger receptor,hsCRP: high sensitive C-reactive protein. Data depicted as median [interquartile range] and analyzed using Mann–Whitney.

|  | Platelet mtDNA content | |
| --- | --- | --- |
|  | R | p-value |
| CD4 NADIR | 0.005 | 0.95 |
| CD4 COUNT | -0.02 | 0.76 |
| CD4/CD8 ratio | 0.03 | 0.68 |
| cART duration | -0.03 | 0.63 |
| HIV infection duration | -0.086 | 0.23 |

Supplemental table 3 Pearson’s correlation coefficient between platelet mtDNA and clinical parameters. mtDNA was normalized using inverse rank-based transformation.

|  | Principal component 1 | |  | Principal component 2 | |
| --- | --- | --- | --- | --- | --- |
|  | R | p-value |  | R | p-value |
| CD4 NADIR | 0.13 | 0.095 |  | 0.09 | 0.24 |
| CD4 COUNT | 0.05 | 0.56 |  | 0.07 | 0.34 |
| CD4/CD8 ratio | 0.09 | 0.26 |  | -0.02 | 0.79 |
| cART duration | -0.09 | 0.24 |  | 0 | 0.96 |
| HIV infection duration | -0.04 | 0.56 |  | 0.02 | 0.83 |
| sCD14 | -0.05 | 0.43 |  | 0.02 | 0.81 |
| sCD163 | -0.1 | 0.15 |  | -0.09 | 0.2 |
| hsCRP | -0.04 | 0.53 |  | 0.01 | 0.88 |

Supplemental table 4 Pearson’s correlations between Principal component 1 and Principal component 2 with clinical variables and inflammation.

**
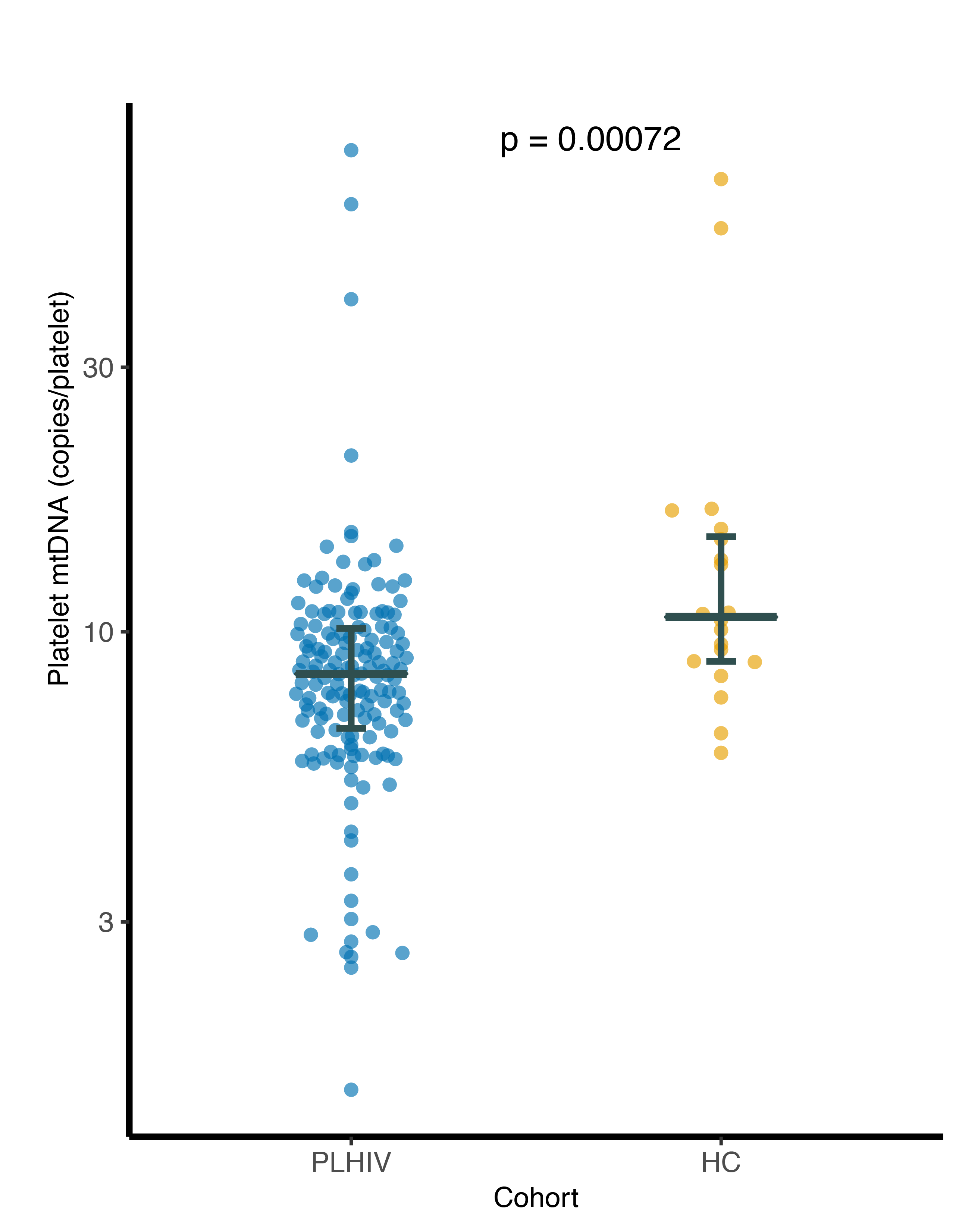
**

**Supplemental Figure 1; Platelet mtDNA levels as copies/platelet.** Subgroup analysis of individuals Age >40 years (people living with HIV (PLHIV): n=173, healthy controls (HC): n=22). Data are shown as dotplot with error bars median and interquartile range (IQR). Data were analyzed using unpaired Student’s T-test. People living with HIV (PLHIV); healthy controls (HC).

Supplemental Figure 2; Platelet mtDNA levels as copies/platelet. A) abacavir-use B) Integrase inhibitor use (INSTI) C) non-nucleoside reverse transcriptase inhibitor use (NNRTI) D) Protease inhibitor use (PI). E) History of Zidovudine (AZT) use. Data were analyzed using Mann–Whitney.

Supplemental Figure 3; Platelet mtDNA levels stratified by use of co-medication. A) antihypertensive drugs (eg. ACE-inhibitor, diuretics) B) low-dose Acetylsalicylic acid (80mg/day; ASA; Aspirin) C) Cholesterol lowering drug class statins D) Anti-diabetics drug Metformin. Data were analyzed using Mann–Whitney.

Supplemental Figure 4; Correlation between inflammation and platelet mtDNA. A) soluble CD14, a marker of in vivo monocyte activation B) high-sensitive C-reactive Protein (hsCRP). sCD14 and hsCRP were log-transformed. mtDNA/pl were transformed using an inverse rank-based normalization. Data were analyzed using Pearson’s coefficient.


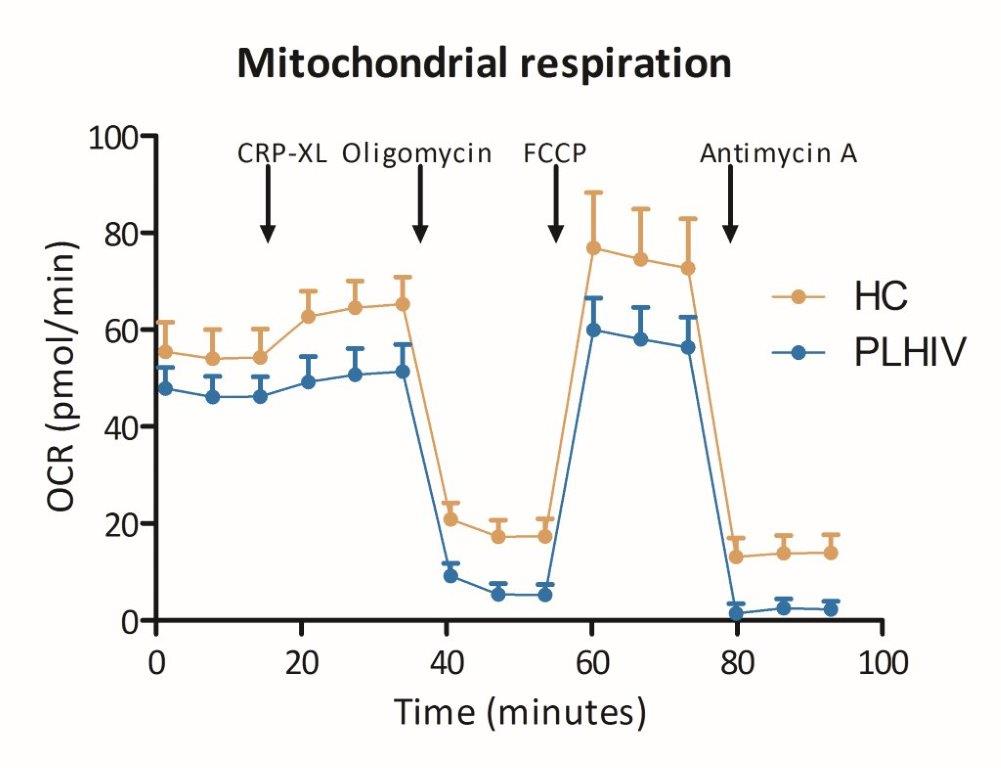


**Supplemental Figure 5; Platelet energy phenotype determined by the Seahorse extracellular flux analyzer after CRP-XL stimulation.** Samples were measured with five replicates, 1.10^7 washed platelets per well. Five age-and sex-matched donors per group. Real-time mitochondrial respiration depicted as oxygen consumption rate (OCR) at 18 timepoints. Four agonists or inhibitors were added in the following order: 1) collagen-related peptide (CRP-XL; 50ng/mL or medium causing platelet activation 2) Oligomycin which inhibits cellular ATP production 3) Carbonyl cyanide-4-(trifluoromethoxy) phenylhydrazone (FCCP), an uncoupling agent causing maximum oxygen consumption through complex IV and 4) Antimycin A which inhibits all mitochondrial respiration (C) real-time glycolysis as extracellular acidification rate (ECAR).

Supplemental Figure 6; Platelet energy phenotype after ex vivo stimulation – Platelet energy phenotype was determined using a Seahorse XFe96 Analyzer. (A) baseline Oxygen consumption rate (OCR) in pmol/min and extracellular acidification rate (ECAR) in mpH/min at baseline. (B) Change in OCR and ECAR after ex vivo stimulation with Trombin (TRAP; 50uM), Collagen-related peptide (CRP-XL; 50ng/mL) or unstimulated. (C) Maximal mitochondrial respiration capacity after FCCP (1μM) stimulation and maximal glycolysis capacity after oligomycin (1μM). (D) Percentage used after ex vivo stimulation of total energy capacity. Data shown are means with ± SEM.

B

A


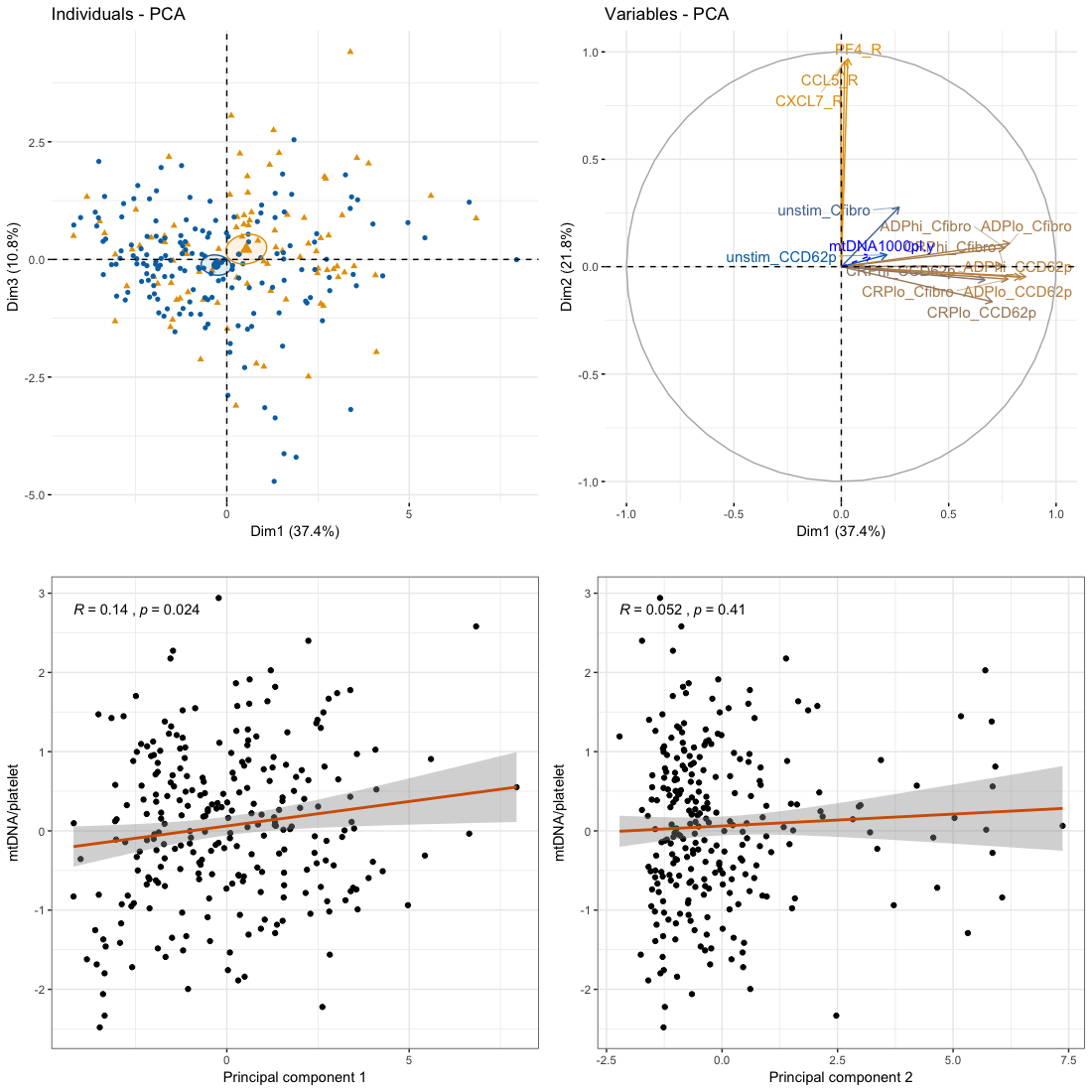


D

C

Supplemental figure 7; Principal component analysis (PCA) of platelet activation and reactivity markers. (A) Coordinates of individuals on PC1 (x-axis) and PC2 (y-axis). All platelet activation markers (plasma levels of CCL5, CXCL7 and PF4; unstimulated P-selectin expression and unstimulated fibrinogen binding) and platelet reactivity markers (P-selectin expression and fibrinogen binding after ex vivo stimulation) were used in this PCA. (B) Figure shows the direction of the used variables. Adenosine diphosphate (ADP), Fibrinogen binding (Fibro), P-selectin (CD62p). (C-D) correlation between PC1/PC2 and mtDNA/pl. Platelet mtDNA (Y-axis) was normalized using inverse rank-based transformation.


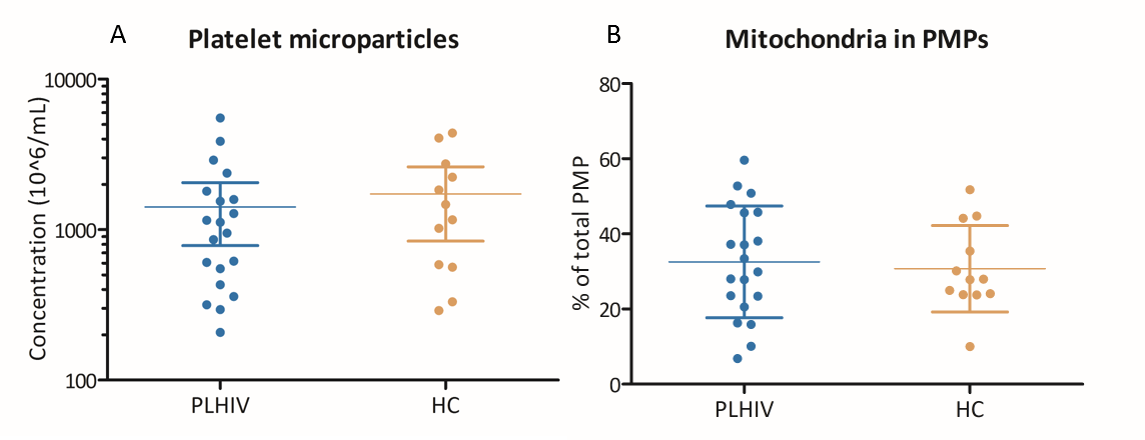


**Supplemental Figure 8 Platelet microparticles and platelet mitochondrial release (A**) Platelet microparticle (PMP) count were determined by CD61, CD41, P-selectin (CD62p) and Annexin V expression and normalized to beads count. (B) The percentage of mitochondria positive PMPs were measured using Mitotracker Green. 17 HIV infected individuals and matched controls were used for this experiment. No significant differences could be observed by using the student’s T-test. Data are depicted as mean ±standard deviation.


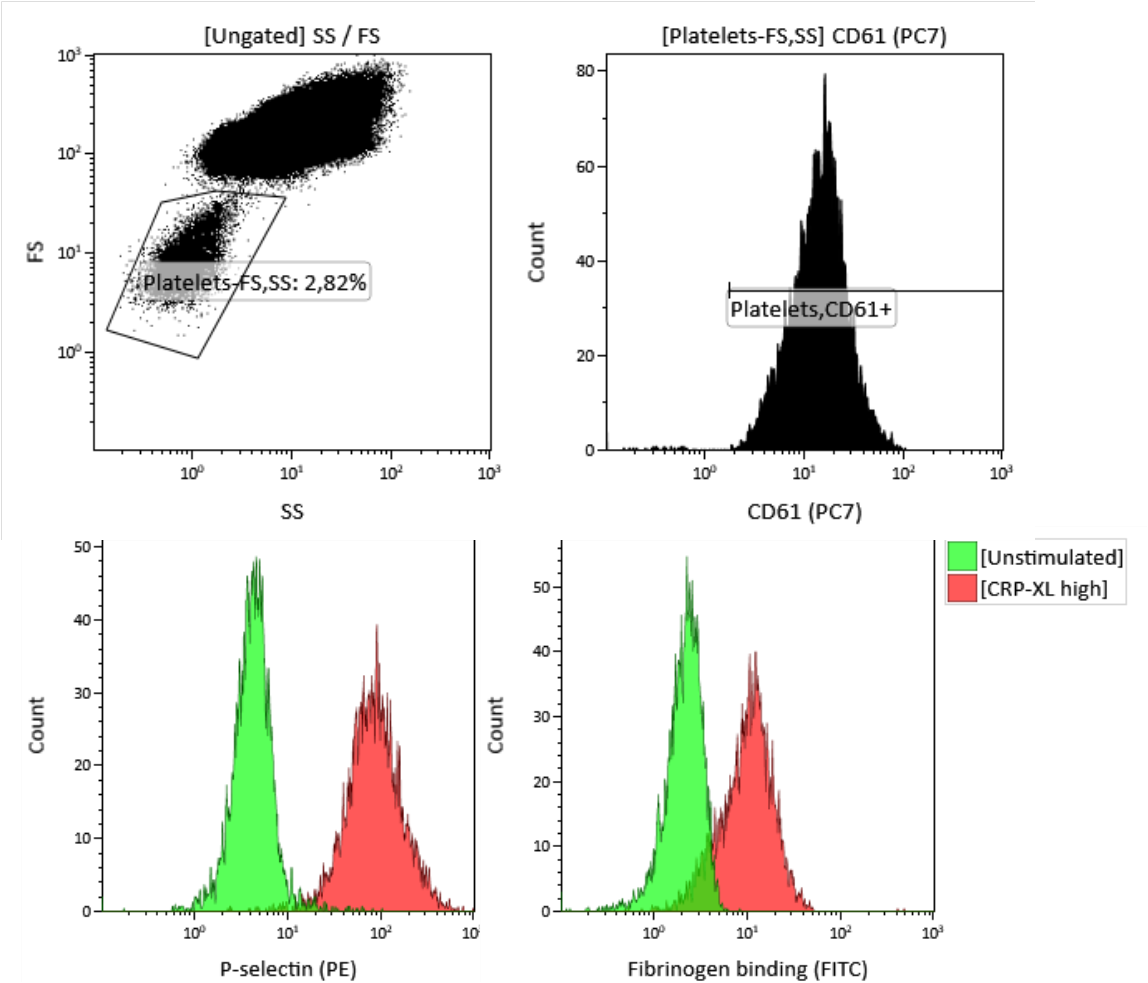


Supplemental Figure 9; Gating strategy using Kaluza 2.1 (Beckman Coulter) for platelet reactivity. First platelets were gated based on size (Forward scatter), granularity (Sideward scatter) and CD61 positivity. Thereafter P-selectin expression and Fibrinogen binding were determined upon stimulation.


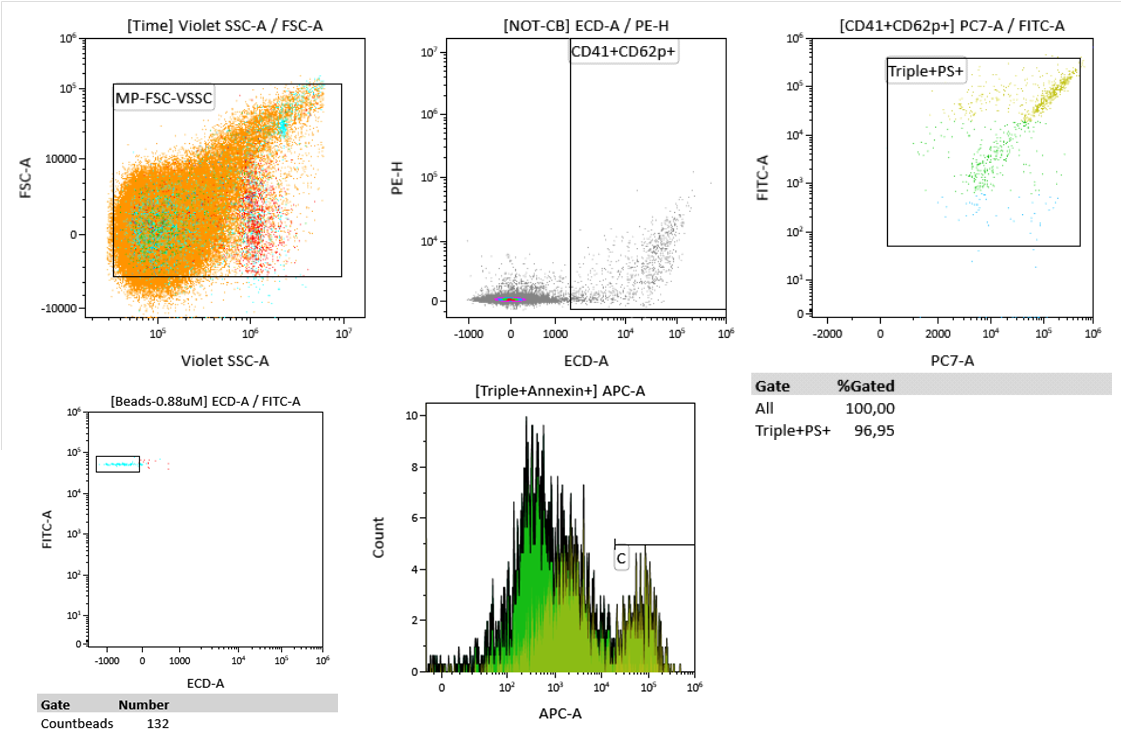


Supplemental Figure 10; Platelet microparticle gating strategy. MPs were labeled with MitoTracker Deep Red (200 nM; Invitrogen, Breda, The Netherlands) in HBS at room temperature containing calcium and subsequently stained with anti-CD41 (Beckman Coulter), anti-CD61 (Biolegend, San Diego, CA), anti-CD62p (Biolegend), Annexin-V (Biolegend) and anti-CD45 (Beckman Coulter). MPs were analyzed using an Cytoflex flow cytometer (Beckman Coulter, Brea, CA) including the sensitive violet Side Scatter (405nM; VSSC) and FSC for detection of ultra-small particles (1µm) (33). Platelet MPs (PMPs) were selected based on aforementioned markers.
